# Network analysis reveals essential proteins that regulate sodium-iodide symporter expression in anaplastic thyroid carcinoma

**DOI:** 10.1038/s41598-020-78574-x

**Published:** 2020-12-08

**Authors:** Hassan Rakhsh-Khorshid, Hilda Samimi, Shukoofeh Torabi, Sayed Mahmoud Sajjadi-Jazi, Hamed Samadi, Fatemeh Ghafouri, Yazdan Asgari, Vahid Haghpanah

**Affiliations:** 1 Department of Biochemistry, Faculty of Biological Sciences, Tarbiat Modares University, Tehran, Iran; 2 Apoptosis Research Centre, National University of Ireland, Galway, Ireland; 3 Endocrinology and Metabolism Research Center, Endocrinology and Metabolism Clinical Sciences Institute, Tehran University of Medical Sciences, Dr. Shariati Hospital, North Kargar Ave, Tehran, 14114 Iran; 4 Department of Stem Cells and Developmental Biology, Cell Science Research Center, Royan Institute for Stem Cell Biology and Technology, Academic Center for Education, Culture and Research (ACECR), Tehran, Iran; 5 Cell Therapy and Regenerative Medicine Research Center, Endocrinology and Metabolism Molecular-Cellular Sciences Institute, Tehran University of Medical Sciences, Tehran, Iran; 6 Department of Biotechnology, Faculty of Life Sciences and Biotechnology, Shahid Beheshti University, Tehran, Iran; 7 Department of Medical Biotechnology, School of Advanced Technologies in Medicine, Tehran University of Medical Sciences, Italia St., Tehran, 1417755469 Iran; 8 Personalized Medicine Research Center, Endocrinology and Metabolism Clinical Sciences Institute, Tehran University of Medical Sciences, Tehran, Iran

**Keywords:** Cancer, Systems biology, Endocrinology

## Abstract

Anaplastic thyroid carcinoma (ATC) is the most rare and lethal form of thyroid cancer and requires effective treatment. Efforts have been made to restore sodium-iodide symporter (NIS) expression in ATC cells where it has been downregulated, yet without complete success. Systems biology approaches have been used to simplify complex biological networks. Here, we attempt to find more suitable targets in order to restore NIS expression in ATC cells. We have built a simplified protein interaction network including transcription factors and proteins involved in MAPK, TGFβ/SMAD, PI3K/AKT, and TSHR signaling pathways which regulate NIS expression, alongside proteins interacting with them. The network was analyzed, and proteins were ranked based on several centrality indices. Our results suggest that the protein interaction network of NIS expression regulation is modular, and distance-based and information-flow-based centrality indices may be better predictors of important proteins in such networks. We propose that the high-ranked proteins found in our analysis are expected to be more promising targets in attempts to restore NIS expression in ATC cells.

## Introduction

Anaplastic thyroid carcinoma (ATC) is a very rare tumor of the thyroid gland, featuring undifferentiated tissue. ATC is diagnosed in about one percent of thyroid cancers with nearly 100 percent disease-specific mortality^1^. In most cases, surgery is intended to prevent imminent airway compromise and despite multimodality approach including post-surgery radio/chemotherapies, many ATC patients have very poor outcome^2^. Radioactive iodine administration which has been routinely applied and considered to be effective in differentiated thyroid cancers, is not successful in ATC patients as ATC tissue does not concentrate iodide due to defects in sodium-iodide symporter (NIS) expression, structure or translocation^3^. Attempts have been made to induce radioactive iodine uptake in ATC cells^4^, e.g., by stable expression of NIS^5^ or suppression of inhibitory signaling pathways^6,7^, however none have yet been clinically used for ATC. The inability of ATC cells to respond to radioactive iodine seems to be the result of several genetic and epigenetic abnormalities. ATC is a genetically complex disease, and various mutations, notably in *BRAF*, *TP53*, *TERT*, *RAS*, and *PIK3CA,* have been noted in samples^8–11^. Moreover, many breaks and copy number variations have been observed in ATC samples^12^. For instance, with regard to NIS expression it was found that BRAF^V600E^, one of the most frequent mutations in ATC, leads to NIS downregulation through induction of TGFβ secretion^13^ or regulation of DNA methyltransferase 1^14^. In addition, membranous NIS-expressing thyroid tumor samples were shown to have wild-type BRAF and N-RAS^15^. Moreover, suppression of MAPK and PI3K/AKT signaling pathways, two pathways with the most frequent mutations in ATC, led to NIS restoration^6,7^. This complicated and multi-pathway regulation of NIS expression has persuaded us to exploit other approaches in order to better understand it.

At several levels, biological entities show such complicated structures and behaviors that systemic approaches are required to complement studies on single molecules. Further encouragement to try these approaches came from the similarities between biological and non-biological (e.g., social) networks that facilitate use of similar methods^16^. Centrality analysis is one of these methods employed by biological researchers to determine the importance of each node (e.g., protein) in a network (e.g., protein–protein interaction (PPI) networks)^17^. In such networks, protein interactions are undirected and unweighted edges^18^. Centrality analysis has been applied in order to find essential proteins of PPI networks in a wide range of organisms^19–23^. This method has also been brought to cancer research^24^. In almost all studies, the goal was finding the most suitable centrality indices or a combination of them, usually by comparing the ranking results within experimental data^17–23,25–28^. Finding essential proteins of a network using centrality analysis can help researchers to design therapeutics and genetic manipulations with a minimalistic approach, and therefore lower cost.

Considering the complexity of NIS expression regulatory pathways in ATC, we attempted to simplify it by building a model network comprising the transcription factors and signaling pathways (MAPK, TGFβ/SMAD, PI3K/AKT, and TSHR) related to NIS expression^13,29,30^. Based on 167 input proteins, the NIS regulatory protein interaction network (NIS-ERPIN) was established. These proteins include transcription factors and signaling pathway proteins that regulate NIS expression, along with some other proteins that interact with them. NIS-ERPIN has then been analyzed for both modularity and centrality and sorted based on different centrality indices. Ranks of proteins in each centrality index have been considered as indicators of proteins essentiality within the network. Protein essentiality in this network may be utilized in order to choose molecular targets for NIS expression and consequently response to radioactive iodine therapy in ATC patients if one is going to disrupt the network.

## Methods

### Creating the NIS-ERPIN

We have built a network of proteins, mainly including the four signaling pathways and transcription factors that regulate NIS expression (Supplementary Table S1). These proteins were initially mined manually through literature review and were enriched by several interacting proteins that were recommended by BioGRID^31^, together making the list of 167 input proteins (Supplementary Figure S1). BioGRID is the Biological General Repository for Interaction Datasets, a curated database of different types of interactions, notably protein–protein interactions. Using Cytoscape (version 3.7.1)^32^, a software capable of modeling the networks of molecular interactions, a network was reconstructed with the data from BioGRID. It is possible to create a network for which all nodes are not necessarily connected to each other. Therefore, we have a whole network including different connected sub-networks. In most cases, there is a very large connected sub-network and other small connected sub-networks. If the other small sub-networks contain a very small number of nodes (compared to the largest one), it is possible to remove them from the analyses. At the present study, the largest connected sub-network was considered for further topological analyses which contains 1,278 nodes and 76,924 edges or interactions (Supplementary Figure S2).

### Centrality analysis

Eleven centrality indices were used in this study: Degree, Betweenness, Closeness, Bottleneck, Radiality, Stress, Clustering Coefficient, EcCentricity, Edge Percolated Component (EPC), Density of Maximum Neighborhood Component (DMNC), and Maximal Clique Centrality (MCC)^33^. Based on the primary ranking, eight centrality indices were informative and thus categorized in three groups: (1) representing the number of immediate neighbors of a node (Degree), (2) indicating the number of interactions in the circle of neighbors (DMNC, and Clustering Coefficient), and (3) reflecting distance and information flow in the network (EPC, Closeness, Radiality, Betweenness, and Stress).

Centrality indices in each category averaged out to give a combination index which we used to compute weighted and unweighted averages of protein ranks. Since we hypothesized that, in NIS-ERPIN, distance- and information flow-based centrality indices are better predictors of protein essentiality, weighted averages were computed to reflect this assumption. The weighted average was calculated by giving a factor of 0.7 to the average of information flow indices, 0.2 to Degree, and 0.1 to the average of Clustering Coefficient and DMNC.

### Modularity analysis

A network of protein interactions was generated using STRING database^34^. The output file, the list of protein interactions, was imported in Gephi 0.9.2^35^ for modularity analysis by Blondel’s method^36^. A graph of 2039 nodes and 76,924 edges was created with the resolution set at one. For comparison, three random graphs with the same number of nodes and resolutions were also generated.

### Mutated genes in ATC

To obtain the mutational landscape of genes that encode proteins of NIS-ERPIN, we used the results of the whole-exome sequencing on ATC samples with the largest sample size, including 22 ATC tissue specimens and four ATC cell lines^37^. At the present study, the list of mutation frequencies for proteins of NIS-ERPIN in ATC is extracted from the results of Kunstman et al.^37^ study and is presented in Supplementary Table S2. The mutation frequencies, normalized and sorted based on the protein length, in ATC were compared with the lists obtained from the centrality analysis. The correlation between lists was evaluated using R software (version 3.5.2) in Mac Operating System.

## Results

### Scoring based on centrality indices and averaging out the proteins ranks

Of the eleven centrality indices used, the results of three (Bottleneck, MCC, and EcCentricity) were not informative (data are not shown) and as a result were excluded from further analyses. They were not informative because their lists consist of mostly repetitive scores. The high-ranked proteins of the eight informative indices are presented in Table 1. Results of the weighted and unweighted averages of protein ranks of three centrality categories are presented in Table 2.

**Table 1:** Top-20 proteins of the eight informative centrality indices.

No	Degree	Betwenness	Closeness	Radiality	Stress	EPC	DMNC	Clustering coefficient
1	AKT1	AKT1	AKT1	AKT1	AKT1	ALB	MAPKAP1	GSX1
2	TP53	TP53	TP53	TP53	PIK3CA	AKT1	IL1A	PTTG1IP
3	GAPDH	SRC	GAPDH	GAPDH	IL6	GAPDH	CASP9	ERRFI1
4	PIK3CA	PIK3CA	PIK3CA	PIK3CA	ALB	PIK3CA	XIAP	CAMP
5	ALB	ALB	ALB	ALB	SRC	JUN	CAMP	FOXE1
6	SRC	IL6	SRC	SRC	TP53	TP53	PARP1	PDK1
7	IL6	MAPK1	IL6	IL6	MAPK1	MAPK3	BCL2L1	MAFA
8	MAPK1	TNF	MAPK1	MAPK1	TNF	PIK3CB	WEE1	SLA
9	MAPK3	EGFR	MAPK3	MAPK3	JAK2	IL6	CHEK1	DAPP1
10	JUN	EGF	JUN	JUN	PIK3CD	PIK3CG	FGF1	MAPKAP1
11	PIK3CG	GAPDH	PIK3CG	PIK3CG	PIK3CB	PIK3CD	MMP2	SLC5A5
12	PIK3CD	MAPK3	PIK3CD	PIK3CD	EGF	MAPK1	RPS6KA1	APEX1
13	TNF	JUN	TNF	TNF	MAPK3	MYC	HIF1A	NKX2-1
14	PIK3CB	VEGFA	PIK3CB	PIK3CB	EGFR	SRC	PPARG	IL1A
15	EGF	PIK3CD	EGF	EGF	PIK3CG	EGFR	DAPP1	HES1
16	EGFR	JAK2	EGFR	EGFR	GAPDH	INS	EGR1	TSHB
17	MYC	PIK3CB	MYC	MYC	VEGFA	TNF	CDC25A	CDC25B
18	VEGFA	PIK3CG	VEGFA	VEGFA	JUN	EGF	EIF4EBP1	THRB
19	INS	MYC	INS	INS	IL2	MAPK8	MDM2	WEE1
20	MAPK8	INS	MAPK8	MAPK8	TGFB1	HRAS	CDC25C	UBTF

**Table 2:** Weighted and unweighted averages of proteins rank after categorizing centrality indices in three groups, as mentioned in “Methods”.

Distance and information flow	Degree	Interaction among neighbors	Unweighted average	Weighted average
AKT1	AKT1	TERF2IP	PARP1	AKT1
PRDM10	PRDM10	CAMP	BCL2L1	PRDM10
TP53	MBOAT4	MAPKAP1	CASP9	IL2
ALB	TP53	IL1A	XIAP	NFKB1
PIK3CA	GAPDH	ERRFI1	FGF1	GAPDH
MBOAT4	PIK3CA	DAPP1	MMP2	MBOAT4
IL6	ALB	XIAP	PPARG	BCL2
SRC	SRC	WEE1	EGR1	TP53
GAPDH	IL6	CASP9	CASP3	IL6
MAPK1	MAPK1	PDK1	HIF1A	ALB
MAPK3	MAPK3	SLA	MDM2	PIK3CA
JUN	JUN	CHEK1	IL1A	JUN
TNF	PIK3CG	APEX1	CDC25A	MTOR
PIK3CD	PIK3CD	CDC25B	RB1	STAT3
PIK3CB	TNF	DDIT3	CDKN1B	PIK3CB
PIK3CG	PIK3CB	PTTG1	IL1B	MAPK3
EGF	EGF	PARP1	RPS6KA1	TNF
EGFR	EGFR	RPS6KA1	PTEN	PIK3CD
MYC	MYC	UBTF	ESR1	PIK3CG
VEGFA	VEGFA	EIF4EBP1	HGF	SRC

### Modularity analysis

**Figure 1:**
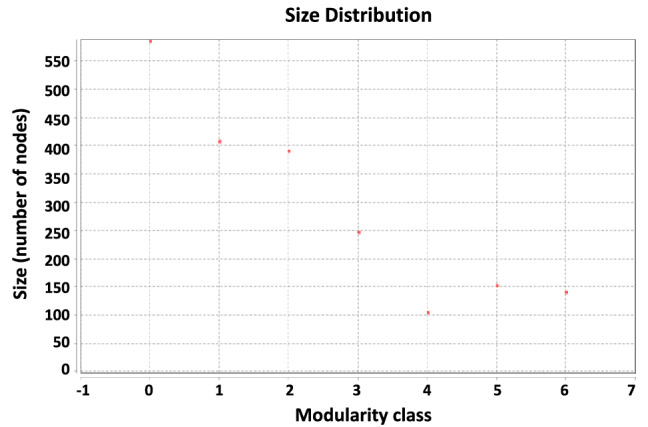
Size distribution of protein communities of NIS expression regulatory network. 7 communities were detected, and the modularity score was calculated 0.411.

The modularity score of 0.411 was calculated for NIS-ERPIN (plot of size distribution is presented in Fig. 1), whereas three random graphs with the same size of nodes had modularity scores of about one fifth (0.085, 0.084 and 0.082; plots of size distribution are presented in Supplementary Figure S3. Moreover, the graph of NIS-ERPIN contained seven communities of nodes, and the random graphs had nine, eight and ten communities, respectively.

### Comparing centrality analysis with mutation frequencies

Interested in the correlation between the rate of mutations and the essentiality of the protein products in NIS-ERPIN, we focused on the mutations of 167 protein-coding genes, reported by Kunstman et al. in the whole-exome sequencing of ATC samples and cell lines^37^. The list of mutations was compared with the lists of protein ranks from centrality analyses. There was no statistically significant correlation between the list of mutation frequencies and any of the centrality indices (Table 3). Also, all correlation coefficients were below 0.3 and considered weak.

**Table 3:** Correlation analysis of mutation rates and centrality indices shows that there is no statistically significant correlation between any of the centrality indices and normalized mutation rates. Mutation data are from a study by Kunstman et al.^37^.

	Betweenness	Closeness	Clustering coefficient	Degree	DMNC	EPC	MNC
Correlation	− 0.0782	− 0.0784	0.1077	− 0.0827	0.1483	− 0.0689	− 0.0827
*P* value	0.3138	0.3122	0.1647	0.2868	0.0551	0.3746	0.2864

	Radiality	Stress	Total average	Group 1 average †	Group 2 average	Group 3 average	Weighted average
Correlation	− 0.0828	− 0.0769	− 0.0369	0.1512	− 0.0765	0.0262	− 0.0644
*P* value	0.2858	0.3217	0.6345	0.0504	0.3241	0.7361	0.4069

^†^Group and weighted averages have been defined as following:

Group 1 average: average of ranks of DMNC and CC.

Group 2 average: average of ranks of closeness, radiality, betweenness, stress and EPC.

Group 3 average: average of ‘Group 1 average', ‘Group 2 average’ and ranks of degree.

Weighted average: average of ‘Group 1 average × 0.1’, ‘Group 2 average ✕ 0.7’ and ranks of ‘degree × 0.2’.

## Discussion

Centrality analysis has been recently explored in thyroid cancer research^38,39^. In doing so, the two groups of Shang et al*.* and Hossain et al*.* first regarded differential gene expression as their criterion for the importance of genes and then built their networks. Taking a different approach here, we have built our network without referring to the gene expression data and therefore we provide a protein interaction network which can be used as a basis for further studies. When creating strategies to disrupt the NIS expression regulatory network, gene expression data can be imported to strengthen or weaken edges and nodes of the network.

An interesting result of our investigation was finding the concentration of apoptotic proteins on top of the unweighted average list of the three centrality categories (Table 2). This outcome indicates that considering all eight centrality indices explored in this study, several apoptosis-related proteins, including BCL2L1, CASP9, XIAP, CASP3, and PARP1, are important in the network. Apoptosis has been proposed as a mechanism of cell death after radioactive iodine uptake in thyroid^40^ and non-thyroid^41–44^ cancer cells. However, our results suggest that apoptosis-related proteins may have a notable role in regulating NIS expression.

Categorizing centrality indices, we hypothesized that those indices, indicating distance and information flow in the network, were better indicators of essentiality in the NIS-ERPIN, if one was going to disrupt the function of the network in ATC. These indices, including Betweenness, Closeness, Stress, Radiality, and EPC, can provide information about the fast flow of signal in the network. Betweenness and Closeness have been shown to present essential proteins of PPI networks in yeast, worm, and fruit fly^22^. Betweenness effectiveness in discriminating essential proteins is proposed to be independent of the number of connections (Degree centrality)^22^. In a homology-based study in yeast PPI network, Xiong et al*.* found that cancer proteins tend to have higher Betweenness scores than average^24^. In a study on prostate cancer, Closeness and Betweenness were found to be more predictive of unknown genes/proteins related to the disease, whereas Degree was able to explore known related genes/proteins accurately^25^. Centrality indices of Degree, Betweenness, and Closeness have been proposed to be profitable in exploring essential proteins of cancer PPI networks^45^ and have been applied in studying the pan-cancer network of proteins related to epithelial-mesenchymal transition^46^. However, we reasoned that Degree centrality could give high scores to more known proteins due to the simple fact that these more recognized proteins have been the focus of more studies, and therefore more interactions (network neighbors) have been found for them. Considering the distance-based indices, we found that many top proteins in these lists are encoded by highly mutated genes in ATC (Table 2). The results of the present study suggest that information-flow- and distance-based centrality indices may be useful in predicting the essential proteins in regulating NIS expression.

To emphasize the importance of distance and information flow in the network, we put more weight on this category when averaging ranks. We found that top-20 positions are concentrated with proteins which have been frequently mutated in ATC, including AKT1, TP53, PIK3CA, PIK3CB, MAPK3, PIK3CD, PIK3CG, SRC, MAPK1, and MYC (Table 2). These are all main proteins of AKT/PI3K or MAPK pathways. The essentiality of distance-based and information-flow-based centrality indices in our study is in accordance with experimental efforts to restore the NIS function. By using small molecules to inhibit MEK, AKT, and histone deacetylase, Liu et al*.* could restore NIS expression and iodine uptake in several non-thyroid cell lines^6^. Also, Hou et al*.* gained similar results upon downregulation of BRAF and AKT in melanoma cells^7^. These observations confirm the significance of MAPK and AKT signaling pathways and epigenetic regulation in NIS expression. PI3K and TGFβ were shown to inhibit radioactive iodine uptake, and one mechanism in the case of TGFβ was NIS repression^47^. In another study to restore iodide uptake, transfection of ATC cells with vector encoding wide-type *TP53* gene was successful to express NIS at the mRNA and protein levels and induce radioactive iodine concentration and eventually cell death^48^. Developing a high-throughput screening, Oh et al*.* recently found a new tyrosine kinase inhibitor leading to MAPK signaling pathway inactivation and NIS expression^49^. Accordingly, we conclude that not only do our results suggest that MAPK and AKT/PI3K pathways may be more important in regulating NIS expression but also, they confirm the importance of distance-based centrality indices in this network. Additionally, other high-ranked proteins in the category of distance-related centrality indices may be interesting subjects of study.

In a modular network, communities of nodes which are densely connected can be found, whereas their connections with nodes of other communities are sparse^36^. Finding evidence of modularity is encouraging, as it suggests that there might be nodes which are highly essential for the integrity of the network, regarded as its weakness. The modularity of NIS-ERPIN emphasizes the significance of centrality analysis presented here. Moreover, we investigated if we can find nodes with high scores in Betweenness and low scores in Degree, which might be the connecting nodes of protein communities^50^. However, we observed that there was no such node, and that there was a very strong correlation between scores of Betweenness and Degree (data are not presented).

Curious about any correlation between centrality ranks of proteins and their rate of mutations, we also compared the results of the only available whole-exome sequencing of ATC samples with our lists of centrality indices and combinatory indices. Such a correlation may indicate the importance of highly mutated proteins for the NIS-ERPIN; however, we found no statistically significant correlation. This lack of meaningful correlation was not unexpected since mutation frequencies found in the whole-exome sequencing were only partially in accordance with mutation frequencies found in target-based sequencing studies^8–12^. Whether this controversial outcome is due to possibly inadequate ATC samples investigated in the whole-exome sequencing will be clarified by further examinations. Therefore, future genetic studies may strengthen or weaken the implications of our centrality analysis.

**Figure 2:**
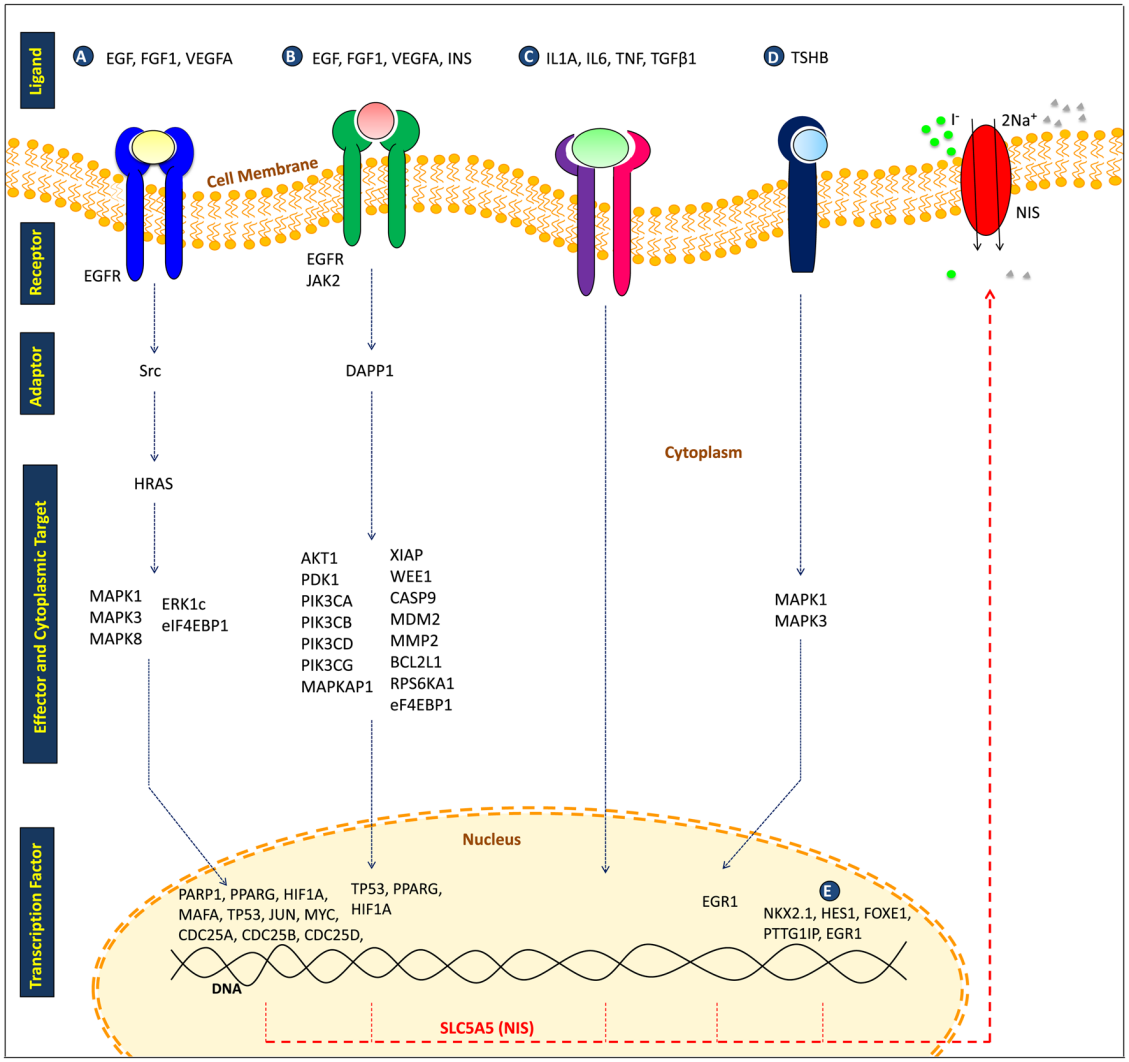
Several proteins of the four signaling pathways, including MAPK (**A**), PI3K/AKT (**B**), TGFβ/SMAD (**C**) and TSH/TSHR (**D**), alongside a few transcription factors (**E**) with a role in the regulation of NIS expression in ATC from the list of 167 input proteins, also appeared in top-20 lists of centrality indices.

Beginning with 167 input proteins to create our network and listing the top-20 in each index, we propose here the essential proteins of the NIS expression regulatory network (Fig. 2). We hypothesized that those centrality indices that represent the distance and flow of information would be better indicators of essential proteins of NIS-ERPIN. We also found that several proteins encoded by highly mutated genes of ATC were high-ranked in these lists. This study can be used to exclude some targets and include other targets in experimental efforts to restore NIS expression. However, we are aware that NIS downregulation is not the only abnormality that results in an inability of thyroid cells to uptake iodide, and that NIS translocation is also worth considering^15^. Nevertheless, some proteins of our network probably have a role in the suppression of NIS function in ways other than expression regulation. For example, it has been shown that β-catenin regulates NIS distribution in thyroid cells^51^. Additionally, NIS inhibitory mutations should be considered, particularly in personalized therapy. In addition to the complicated manner of NIS suppression, it is worth noting that, to interpret raw data from the network analysis, careful reflection on biological context and/or experimental data is required. Besides, there are some limitations regarding the centrality analysis. One of them is different indices could lead to difference on the most important vertex in a network. Hence , using a proper index depends heavily on the context of the reconstructed network and should be chosen with a reasonable process. Another limitation is whether a combination of different indices is more useful than considering an index alone. However, this issue also depends on the basics and properties of the reconstructed network. Therefore, one must find or suggest the proper indices in the study based on the context of the biological problem in the study^52^. Moreover, PPI networks are affected by false positives and, have also not yet been completed^45^, which is a source of inaccuracy in these analyses. Therefore, a more cautious approach to network analysis results is recommended.

## Conclusion

The results of our modularity and centrality analyses suggest a combination therapy approach to induce NIS expression in ATC cases, particularly if the targets are essential proteins we present in this study. We propose several genes/proteins in the NIS expression regulatory network as interesting targets for manipulation, including: AKT1, TP53, PIK3C, MAPK1, MAPK3, SRC, MYC, EGR1, XIAP, BCL2L1, CASP3, and CASP9. Also, several previously unnoticed proteins which rank high in our analyses might prove to be interesting targets of study. Moreover, we presume that lower rank proteins of our weighted and unweighted combinatory indices are of little importance and interest, and that manipulations based on them will probably result in failure due to the fact that they are not essential enough in the NIS expression regulatory network.

## Supplementary information

Supplementary Information.

## Data Availability

Data are available upon request from corresponding authors.

## Abbreviations

AKT1: AKT Serine/Threonine Kinase 1
ATC: Anaplastic Thyroid Carcinoma
BCL2L1: B Cell Lymphoma-2-Like 1
BioGRID: Biological General Repository for Interaction Datasets
BRAF: B-Raf Proto-Oncogene
CASP3: Cysteine-ASPartic Protease 3
CASP9: Cysteine-ASPartic Protease 9
DMNC: Maximum Neighborhood Component
EGR1: Early Growth Response Protein 1
EPC: Edge Percolated Component
N-RAS: NRAS Proto-Oncogene
MAPK1: Mitogen-Activated Protein Kinase 1
MCC: Maximal Clique Centrality
MEK: Mitogen-Activated Protein Kinase Kinase
MYC: MYC Proto-Oncogene
NIS: Sodium-Iodide Symporter
NIS-ERPIN: NIS regulatory protein interaction network
PARP1: Poly [ADP-Ribose] Polymerase 1
PI3K: Phosphoinositide 3-Kinase
PIK3CA: Phosphoinositide 3-Kinase Catalytic subunit A
PIK3CB: Phosphoinositide 3-Kinase Catalytic subunit B
PIK3CD: Phosphoinositide 3-Kinase Catalytic subunit D
PIK3CG: Phosphoinositide 3-Kinase Catalytic subunit G
PPI: Protein–Protein Interaction
SMAD: Sma- And Mad-Related Protein
SRC: SRC Proto-Oncogene
TERT: Telomerase Reverse Transcriptase
TGFβ: Transforming Growth Factor β
TP53: Tumor Protein 53
TSHR: Thyroid-Stimulating Hormone Receptor
XIAP: X-linked Inhibitor of Apoptosis Protein
