# Supplementary material for: Network analysis reveals essential proteins that regulate sodium-iodide symporter expression in anaplastic thyroid carcinoma
Source: Sci Rep. 2020 Dec 8;10:21440. doi: 10.1038/s41598-020-78574-x (PMC7722919; doi:10.1038/s41598-020-78574-x)
Supplement: Supplementary file 1 — Supplementary Information. [file 41598_2020_78574_MOESM1_ESM.pdf]

Network analysis reveals essential proteins that regulate sodium-iodide symporter expression in anaplastic thyroid carcinoma

Hassan Rakhsh-Khorshid[1,2], Hilda Samimi[3], Shukoofeh Torabi[4], Sayed Mahmoud Sajjadi-Jazi[5,3], Hamed Samadi[3], Fatemeh Ghafouri[6,3], Yazdan Asgari[7,*], Vahid Haghpanah[3,8,*]

1 Department of Biochemistry, Faculty of Biological Sciences, Tarbiat Modares University, Tehran, Iran
2 Apoptosis Research Centre, National University of Ireland, Galway, Ireland
3 Endocrinology and Metabolism Research Center, Endocrinology and Metabolism Clinical Sciences Institute, Tehran University of Medical Sciences, Tehran, Iran
4 Department of Stem Cells and Developmental Biology, Cell Science Research Center, Royan Institute for Stem Cell Biology and Technology, Academic Center for Education, Culture and Research (ACECR), Tehran, Iran
5 Cell Therapy and Regenerative Medicine Research Center, Endocrinology and Metabolism Molecular-Cellular Sciences Institute, Tehran University of Medical Sciences, Tehran, Iran
6 Department of Biotechnology, Faculty of Life Sciences and Biotechnology, Shahid Beheshti University, Tehran, Iran
7 Department of Medical Biotechnology, School of Advanced Technologies in Medicine, Tehran University of Medical Sciences, Tehran, Iran
8 Personalized Medicine Research Center, Endocrinology and Metabolism Clinical Sciences Institute, Tehran University of Medical Sciences, Tehran, Iran

* **Corresponding authors**:
Vahid Haghpanah, MD MPH PhD
Endocrinology and Metabolism Research Center,
Dr. Shariati Hospital, North Kargar Ave., Tehran 14114, Iran
Tel: +98 21 88220037-8, Fax: +98 21 88220052
Email: v.haghpanah@gmail.com, vhaghpanah@tums.ac.ir

Yazdan Asgari, PhD
School of Advanced Technologies in Medicine,
Italia St., Tehran 1417755469, Iran
Tel: +98 21 43052000 (Ext: 126), Fax: +98 21 88991117
Email: yasgari@tums.ac.ir

This PDF file includes:

Supplementary Figures S1 - S3

Supplementary Tables S1 and S2

Supplementary Figure S1. Initial network of input proteins and their interactions (including four signaling pathways and transcription factors which regulate NIS expression)

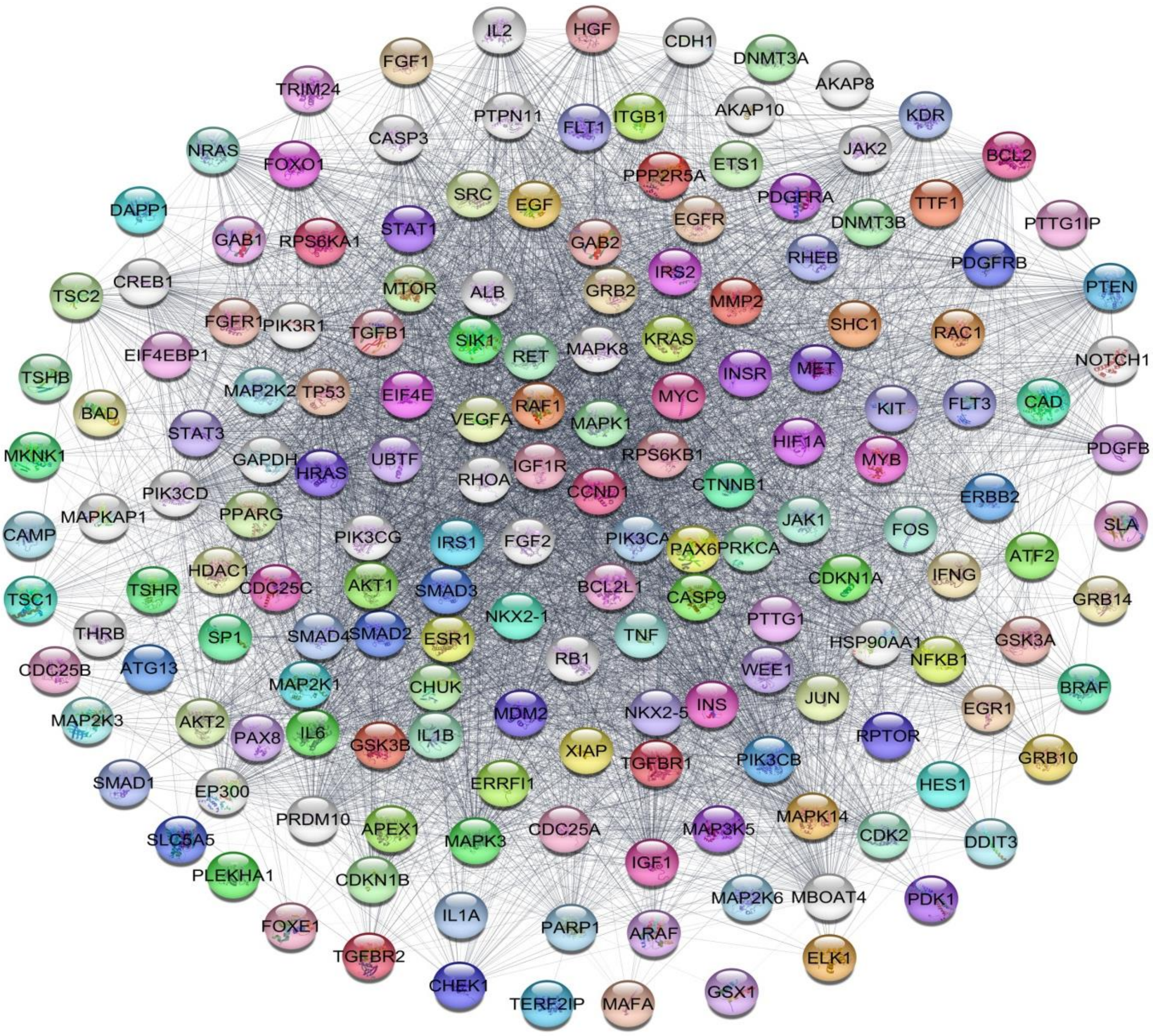

Supplementary Figure S2. Extended network based on the input proteins and their interactions including 1,278 nodes and 76,924 edges

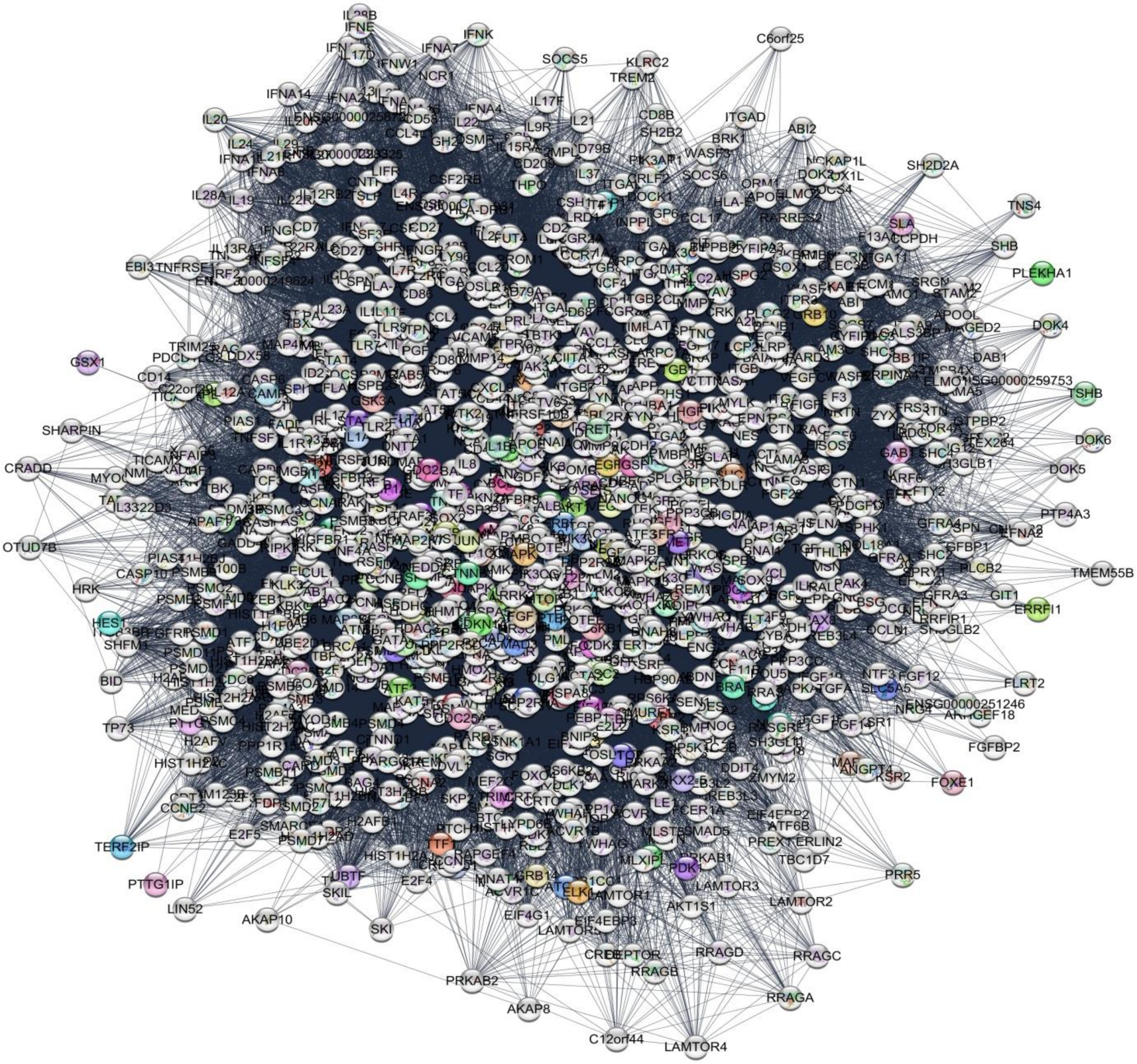

Supplementary Figure S3. Plots of size distribution of three random graphs generated for comparison with NIS-ERPIN.

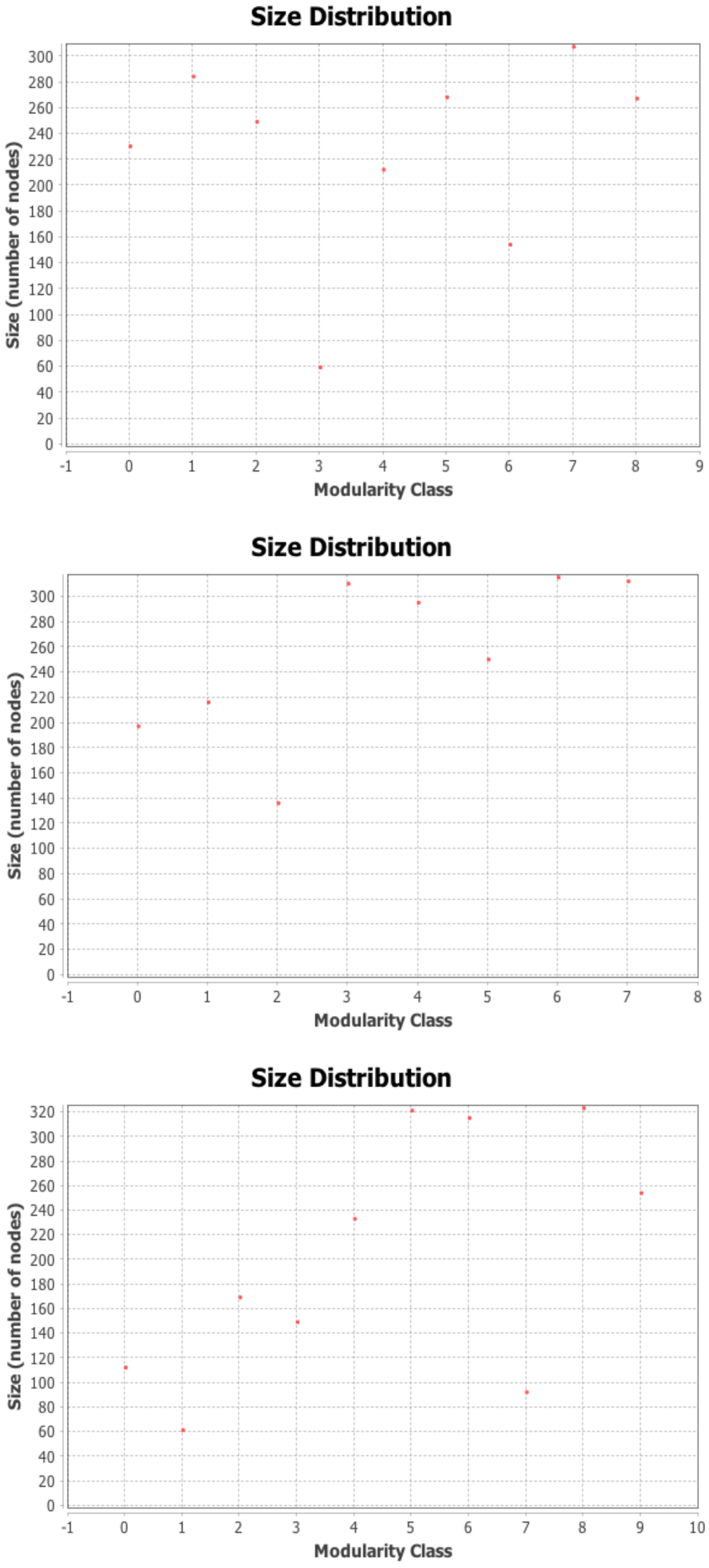

Supplementary Table S1. Input proteins used in creating the network are presented along with their Gene ID.

Name	Gene ID	Name	Gene ID	Name	Gene ID	Name	Gene ID	Name	Gene ID	Name	Gene ID
AKAP10	11216	*CTNNB1*	1499	*GRB14*	2888	*KRAS*	3845	*PDGFB*	5155	*SIK1*	150094
AKAP8	10270	*DAPP1*	27071	*GRB2*	2885	*MAFA*	389692	*PDGFRA*	5156	*SLA*	6503
AKT1	207	*DDIT3*	1649	*GSK3A*	2931	*MAP2K1*	5604	*PDGFRB*	5159	*SLC5A5*	6528
AKT2	208	*DNMT3A*	1788	*GSK3B*	2932	*MAP2K2*	5605	*PDK1*	5163	*SMAD1*	4086
ALB	213	*DNMT3B*	1789	*GSX1*	219409	*MAP2K3*	5606	*PIK3CA*	5290	*SMAD2*	4087
APEX1	328	*EGF*	1950	*HDAC1*	3065	*MAP2K6*	5608	*PIK3CB*	5291	*SMAD3*	4088
ARAF	369	*EGFR*	1956	*HES1*	3280	*MAP3K5*	4217	*PIK3CD*	5293	*SMAD4*	4089
ATF2	1386	*EGR1*	1958	*HGF*	3082	*MAPK1*	5594	*PIK3CG*	5294	*SP1*	6667
ATG13	9776	*EIF4E*	1977	*HIF1A*	3091	*MAPK14*	1432	*PIK3R1*	5295	*SRC*	6714
BAD	572	*EIF4EBP1*	1978	*HRAS*	3265	*MAPK3*	5595	*PLEKHA1*	59338	*STAT1*	6772
BCL2	596	*ELK1*	2002	*HSP90AA1*	3320	*MAPK8*	5599	*PPARG*	5468	*STAT3*	6774
BCL2L1	598	*EP300*	2033	*IFNG*	3458	*MAPKAP1*	79109	*PPP2R5A*	5525	*TERF2IP*	54386
BRAF	673	*ERBB2*	2064	*IGF1*	3479	*MBOAT4*	619373	*PRDM10*	56980	*TGFB1*	7040
CAD	790	*ERRFI1*	54206	*IGF1R*	3480	*MDM2*	4193	*PRKCA*	5578	*TGFBR1*	7046
CAMP	820	*ESR1*	2099	*IL1A*	3552	*MET*	4233	*PTEN*	5728	*TGFBR2*	7048
CASP3	836	*ETS1*	2113	*IL1B*	3553	*MKNK1*	8569	*PTPN11*	5781	*THRB*	21834
CASP9	842	*FGF1*	2246	*IL2*	3558	*MMP2*	4313	*PTTG1*	9232	*TNF*	7124
CCND1	595	*FGF2*	2247	*IL6*	3569	*mTOR*	2475	*PTTG1IP*	754	*TP53*	7157
CDC25A	993	*FGFR1*	2260	*INS*	3630	*MYB*	4602	*RAC1*	5879	*TRIM24*	8805
CDC25B	994	*FLT1*	2321	*INSR*	3643	*MYC*	4609	*RAF1*	5894	*TSC1*	7248
CDC25C	995	*FLT3*	2322	*IRS1*	3667	*NFKB1*	4790	*RB1*	5925	*TSC2*	7249
CDH1	999	*FOS*	2353	*IRS2*	8660	*NKX2-1*	7080	*RET*	5979	*TSHB*	7252
CDK2	1017	*FOXE1*	2304	*ITGB1*	3688	*NKX2-5*	1482	*RHEB*	6009	*TSHR*	7253
CDKN1A	1026	*FOXO1*	2308	*JAK1*	3716	*NOTCH1*	4851	*RHOA*	387	*UBTF*	7343
CDKN1B	1027	*GAB1*	2549	*JAK2*	3717	*NRAS*	4893	*RPS6KA1*	6195	*VEGFA*	7422

CHEK1	1111	*GAB2*	9846	*JUN*	3725	*PARP1*	142	*RPS6KB1*	6198	*WEE1*	7465
CHUK	1147	*GAPDH*	2597	*KDR*	3791	*PAX6*	5080	*RPTOR*	57521	*XIAP*	331
CREB1	12912	*GRB10*	2887	*KIT*	3815	*PAX8*	7849	*SHC1*	6464		

Supplementary Table S2. Missense and nonsense mutations in input proteins of the network have been investigated, and in addition to mutations found in the four commonly used ATC cell lines, were used to calculate normalized mutation rates. Data have been obtained from a whole-exome sequencing studied by Kunstman *et al.* (2015).

Gene Symbol	Total Number of variants	Number of missense/ nonsense mutations	Mutations in 22 tissue samples + cell lines	Protein length (a.a.)	Normalized mutation rates (tissue samples + cell lines)
TP53	6	6	10	346	0.0289017341040462
BRAF	6	6	9	766	0.0117493472584856
NRAS	3	3	4	189	0.0211640211640212
EGF	2	2	3	1165	0.0025751072961373
KRAS	2	2	2	189	0.0105820105820106
PPP2R5A	0	0	2	486	0.0041152263374485
SP1	1	1	2	785	0.0025477707006369
PIK3CA	2	2	2	1068	0.0018726591760299
PRDM10	1	1	2	1156	0.0017301038062283
TSC1	0	0	2	1164	0.0017182130584192
CAD	1	1	2	2225	0.0008988764044943
mTOR	2	2	2	2549	0.0007846214201647
DDIT3	1	1	1	169	0.0059171597633136
HRAS	1	1	1	189	0.0052910052910052
CDKN1B	1	1	1	198	0.0050505050505050
RAC1	1	1	1	211	0.004739336492891
MAP2K6	1	1	1	334	0.0029940119760479
ATF2	1	1	1	374	0.0026737967914438
MAPK3	1	1	1	379	0.0026385224274406
PAX8	1	1	1	398	0.0025125628140703
PLEKHA1	1	1	1	404	0.0024752475247524
AKT2	1	1	1	419	0.0023866348448687
PAX6	1	1	1	436	0.0022935779816513
ERRFI1	1	1	1	462	0.0021645021645021
MAPKAP1	1	1	1	522	0.0019157088122605
GRB14	0	0	1	540	0.0018518518518518
TGFBR2	0	0	1	567	0.0017636684303351
RAF1	1	1	1	648	0.0015432098765432
TSHR	1	1	1	764	0.0013089005235602
CTNNB1	1	1	1	781	0.0012804097311139

SIK1	0	0	1	783	0.0012771392081736
RET	1	1	1	1072	0.0009328358208955
PDGFRB	0	0	1	1106	0.0009041591320072
ERBB2	1	1	1	1225	0.0008163265306122
FLT1	1	1	1	1338	0.0007473841554559
IGF1R	1	1	1	1367	0.0007315288953913
TSC2	0	0	1	1807	0.0005534034311012
